# Excision of Superior Maxillary

**Published:** 1872-10

**Authors:** John P. Wall

**Affiliations:** Tampa, Florida


### EXCISION OF SUPERIOR MAXILLARY.

BY JOHN P. WALL, M.D., TAMPA, FLORIDA,

Louisa R., æt. eight years, was brought to me in June, 1872, with a bony tumor, of slight elevation, of the left cheek. It had first been noticed in the latter part of April preceding, as a hard elevation at the side of the nose. The child was tolerably well grown for her age—had always been healthy, and had not complained of the face either before or after the commencement of the growth. On examination, the tumor was found of a firm, hard consistence, devoid of crackling or fluctuation; firm pressure occasioned some pain. The roof of the mouth was also bulged downward on the left side. An exploratory puncture with a drill was made from the socket of the left canine tooth in the direction of the antrum, and also an incised puncture through the bulging roof; but without any positive results, save to show great vascularity of the bony structure. Being convinced that the bone was diseased, I determined, however, to watch the case for some time, and consequently had the child taken back home, with instructions to bring her in again in the course of a month or so, if the growth appeared to increase.

In the latter part of July, she was brought in again. The tumor now involved the whole of the bone below the orbit, the alveolar process being at least twice as thick as on the right side, and the left incisors were longer than the right. In consultation with Dr. F. Branch, an old practitioner, the removal of the bone was determined upon.

On the 8th of August, with the assistance of Drs. Branch and Wells, and Dr. McMullen (dental surgeon), I proceeded to operate. The patient having been placed on the table, with the head well elevated, chloroform was administered by Dr. Branch; and as soon as anæsthesia was sufficiently induced, the left middle incisor was extracted. A curvilinear incision was made from near the external canthus of the left eye into the angle of the mouth. The facial and two other smaller arteries were tied, and the ligatures cut short. Another incision from near the internal canthus was carried down the side of the nose, around the ala to the mesial line, thence through the lip into the mouth. This cheek flap was dissected close to the bone up to the border of the orbit. The ala and mucous membrane of the nostril were detached, and the mucous membrane of the roof of the mouth divided along the mesial line. A Hey's saw was used to divide the malar and alveolar processes, and to groove the bone at the border of the orbit. The palatal arch and nasal process were divided with bone forceps, and the further bony severance was completed with the chisel and mallet. The remaining soft structures were divided with the knife, in the act of removing the bone. The division of the bone having been effected beyond the limits of the disease, the hemorrhage was trifling, no vessel requiring ligature. After a delay of a few minutes, two pledgets of lint, wet in a saturated solution of persulphate of iron, were introduced against the bony surfaces, the cheek flap brought down and secured along the lines of both incisions with the twisted suture. For this purpose, small cambric-needles were used, both ends being broken off after the application of the thread.

The operation was necessarily protracted and tedious, in consequence of being unable to keep up the continuous administration of chloroform. Considerable blood was swallowed, finally occasioning vomiting, and much care was requisite throughout to avoid strangling by blood. The patient, though much exhausted, stood the operation better than I expected for one of her age. After being put to bed, small and repeated doses of laudanum were given every hour, to relieve nervousness and pain, and to equalize the circulation. This having been accomplished, morphine was afterward used throughout the treatment, to relieve pain and procure sleeep. Inflammatory fever set in on the third day, subsiding in about thirty-six hours, with suppuration. The needles were removed on the fifth day, union having occurred in both incisions, which were afterward supported with adhesive plaster. Lint, wet in a solution of carbolic acid, was introduced into the cavity, and this repeated several times in the twenty-four hours. Twelve grains of quinine were given on the fifth day. Henceforward the case progressed favorably, and the patient was able to return home the eleventh day after the operation, when she rapidly got well.

The bone, after removal, appeared to be softer and more spongy than natural, with a deep-red color. Its resistance was feeble, being easily cut or torn. There appeared to be no disease of the teeth, or of the young gums imbedded in the bone at the roots of the bicuspids; the canine of the second dentition was just cutting through the gum. At no time previous to the operation was there any enlargement of the submaxillary or cervical glands. The chief interest of the case consists in the absence of any kind of pain, the rapidity of the growth without known cause, and the rarity of such a diseased condition of this bone in children.

